# A biogeographic 16S rRNA survey of bacterial communities of ureolytic biomineralization from California public restrooms

**DOI:** 10.1371/journal.pone.0262425

**Published:** 2022-01-14

**Authors:** Kahui Lim, Matthew Rolston, Samantha Barnum, Cara Wademan, Harold Leverenz

**Affiliations:** 1 Department of Civil and Environmental Engineering, University of California at Davis, Davis, California, United States of America; 2 Host Microbe Systems Biology Core Facility, Department of Medical Microbiology & Immunology, University of California at Davis, Davis, California, United States of America; 3 Real-time PCR Research & Diagnostics Core Facility, Department of Medicine & Epidemiology, University of California at Davis, Davis, California, United States of America; Universita degli Studi di Milano-Bicocca, ITALY

## Abstract

In this study, we examined the total bacterial community associated with ureolytic biomineralization from urine drainage systems. Biomineral samples were obtained from 11 California Department of Transportation public restrooms fitted with waterless, low-flow, or conventional urinals in 2019. Following high throughput 16S rRNA Illumina sequences processed using the DADA2 pipeline, the microbial diversity assessment of 169 biomineral and urine samples resulted in 3,869 reference sequences aggregated as 598 operational taxonomic units (OTUs). Using PERMANOVA testing, we found strong, significant differences between biomineral samples grouped by intrasystem sampling location and urinal type. Biomineral microbial community profiles and alpha diversities differed significantly when controlling for sampling season. Observational statistics revealed that biomineral samples obtained from waterless urinals contained the largest *ureC/*16S gene copy ratios and were the least diverse urinal type in terms of Shannon indices. Waterless urinal biomineral samples were largely dominated by the Bacilli class (86.1%) compared to low-flow (41.3%) and conventional samples (20.5%), and had the fewest genera that account for less than 2.5% relative abundance per OTU. Our findings are useful for future microbial ecology studies of urine source-separation technologies, as we have established a comparative basis using a large sample size and study area.

## Introduction

Source-separation technologies such as waterless urinals and low-flow urinals save water and, in the case of waterless urinals, require less plumbing than conventional flush systems. Most urine drainage/collection systems are susceptible to microbially-induced precipitation, or biomineralization. Additionally, lower flow rates associated with water conserving urinals have been implicated in drainage and aesthetic problems. Biomineralization in urine drainage systems has been described as both a viscous sludge that forms at pipe inverts and storage tanks, or as solid formations that are caused by precipitation directly on the pipe wall [1–3]. Ureolytic biofilm and biomineral formations are persistent complications in cleaning and maintenance measures for urine source-separation systems. These biofilm and biomineral formations are why waterless urinals continue to clog despite strong acid and caustic treatments tried in past field studies [4].

Biomineralization is typically a mixed composition of struvite, calcium phosphate, calcium oxalate, calcium carbonate, and organic matter. Its occurrence in source-separation fixtures compromises the social acceptability of the technology due to associations with clogging, odor, and overall user dissatisfaction [4]. While biomineralization in urinals is due to a combination of various mechanisms, a key contributor to its formation is the hydrolysis of urea, or ureolysis, catalyzed by the microbial enzyme, urease. The prevalence, mineral composition, and formation of ureolytic crystallization is a function of urine chemistry such as the pH and ionic strength, modulators and inhibitors of crystallization, and the bacterial community [5–10]. Though ammonification of proteins and dissimilatory nitrate reduction to ammonia contributes to pH increases, ureolysis has been considered the most efficient pathway to raise pH into the range required in biomineral formation [11–15]. Ureolysis involves the production of ammonium and carbonate from the breakdown of urea, leading to an increase in saturation indices that coincide with the pH shift that culminate as biomineral formation [5]. Over time, as the biomineral hardens and accumulates, the pipes will clog in a manner similar to urological catheters [5, 16, 17]. Due to its role in biomineralization, the activity of urease was of particular interest in this study.

This is the first study on the microbial ecology of the biomineralization taken from dedicated urine drainage systems behind urinals. Whereas struvite precipitates studied by Lahr et al. (2016) were produced *in vitro*, the biomineral precipitates examined in this study were taken directly from urine drainage systems in operation along busy California highways. Earlier, Lahr et al. (2016) attempted to characterize the microbial community in urine collected from public events using portable toilets, and found that aged, hydrolyzed urine has less OTU richness than does freshly collected urine (< 24 hours). Lahr et al. (2016) also made observations that bacterial communities associated with artificially induced struvite precipitates did not differ from liquid associated communities. However, no statistical analysis was performed on this empirical observation. Our large sample size study also builds upon the research of Lahr et al. (2016), exploring how differences in urine collection systems, geography, and seasonality can influence the composition of the bacterial communities that are associated with biomineralization. Of note, this novel study also builds upon our previous regression study from the same sampling periods and used the same dataset [18]. Whereas our previous regression study focused solely on predicting the influence of environmental variables on biomineral urease activity, this study substantially differs as it draws new conclusions on the microbial ecology of biomineral samples using different statistical techniques applied to sequencing data [18].

Through a state-wide biogeographic survey using high-throughput 16S rRNA gene sequencing, the objectives of this microbial ecology study were to (a) assess the alpha and beta diversities of biomineral and liquid associated bacterial communities taken from urine drainage systems across California, (b) determine the average bacterial community structure with regard to urinal type (waterless, low-flow, and conventional), and (c) observe the effects of seasonality and intrasystem sampling location on the bacterial community diversity of biomineral samples, and (d) posit the relationship between alpha diversity metrics and the abundance of the *ureC* gene estimated using quantitative polymerase chain reaction (qPCR).

## Materials and methods

In 2019, ureolytic biomineralization and urine from 11 public restrooms owned by the California Department of Transportation (Caltrans; funding agency) were sampled for a microbial ecology study using high throughput sequencing of the 16S rRNA marker. These public rest areas were chosen because they were known by Caltrans to be frequently clogged due to ureolytic biomineral precipitates. Permission to sample these rest areas was obtained via the funding contract (Agreement Number 65A0734) with the sponsoring agency. All environmental metadata including sampling dates can be found at the Dryad repository (DOI: 10.25338/B82906). Nucleotide sequences were deposited at the National Center for Biotechnology Information (NCBI) Sequencing Read Archive (SRA) under the BioProject Accession number PRJNA699694.

### Sample collection

**Fig 1:**
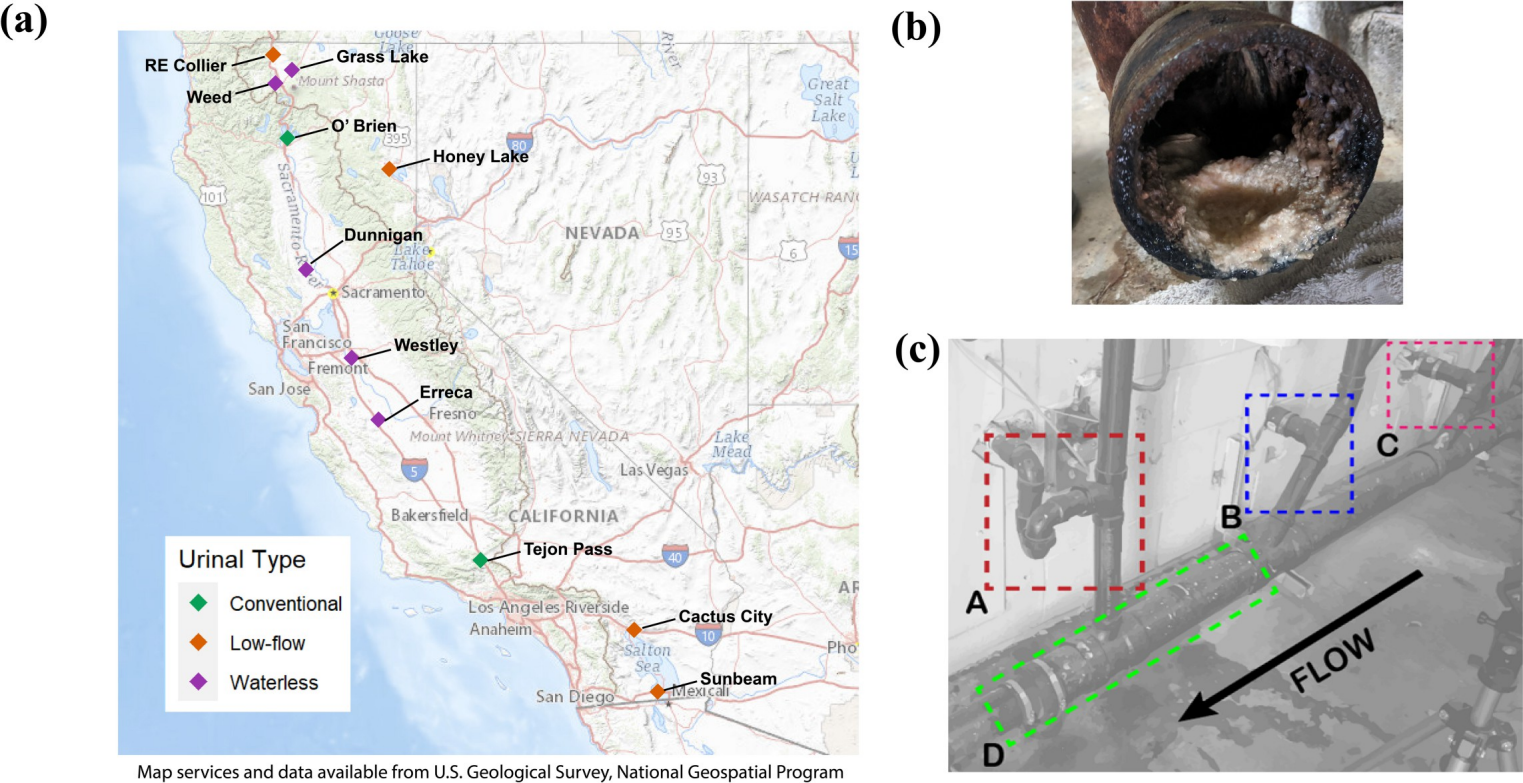
Sampling sites, characteristic sample, and representative intrasystem sampling locations in the plumbing gallery. In total, (a) 11 separate rest areas owned by the California Department of Transportation were sampled for this study, (b) characteristic biomineral formation on the invert of a gallery main drain at Erreca northbound rest area. (c) the gallery drainpipes directly succeeding the urinals such as that shown from Erreca northbound rest area were typical sampling sites. As depicted are: (A) sink (not sampled), (B) American Disability Act (ADA) drain line, (C) standard urinal height drain line, (D) main drain.

A total of 2 conventional, 4 low-flow, and 5 waterless urinals located at public safety roadside rest areas (SRRAs) were observed in this study as mapped in Fig 1. Conventional rest areas are those fitted with urinals producing ~ 1 gal/flush, low-flow ~0.125 gal/flush, and waterless no flush. If there was sufficient mass available, biomineralization deposits were scraped into sterile 50 mL conical tubes from fouled fixtures, cartridges, drain traps, screens, and drain lines such as those shown in S1 Fig from the S1 File.

All samples were stored in an ice chest immediately after collection and processed immediately after return to campus. Previous work monitoring the ureolysis rate in soils have found that a distinct slowdown in ureolytic rate was not detected until 8 months of storage and is consistent with a past study on the effects of storage on soil urease activity [19]. Regarding the effects of storage condition on soil microbial community compositions, others have demonstrated that neither storage time nor storage temperature substantially altered overall communities relative to more than 500 previously examined soil samples [20]. DNA sequencing reads are shown to be consistent for samples stored at both 4°C and 20°C for 0, 2, and 5 days prior to freezing as shown in the S2 Fig from the S1 File. As such, the sampling preservation measures were deemed adequate. Therefore, we demonstrated adequate storage techniques during the sampling trips by evaluating the effects of time and temperature on the observed community structure.

### Biomineral ureolytic enzyme activity characterization

Biomineral ureolytic enzyme activity was measured for correlation analyses with alpha-diversity scores. To determine the enzymatic activities of biomineral sample, a known wet mass of the biomineral samples was suspended and mixed in a 100 mL volume of 7.3 pH 200 mM (4-(2-hydroxyethyl)-1-piperazineethanesulfonic acid) (HEPES) buffer containing 2.5% urea m/m. The rate of increase in conductivity is proportional to that of urea hydrolysis and can be used as a surrogate measure for enzymatic activity [21]. As a comparative basis between samples, one unit of specific activity is defined as the uS cm^-1^ per gram of volatile solids (VS). Volatile solids were determined using standard methods for examination of water and wastewater [22].

### Quantifying *ureC* and 16S gene abundance using real-time polymerase chain reaction (qPCR)

To examine the relationship between the biomineral bacterial community structure, diversity, and ureolytic genes, the genomes representing the presence of urease genes were examined by qPCR. A similar protocol was previously described [23, 24]. The urease associated gene and 16S assays were designed on the urease alpha subunit encoding gene (*ureC*) and the 16S rRNA gene, respectively. Primer sequences were obtained from the literature [23, 24].

Biomineral samples were kept frozen at -20°C prior to DNA extraction. DNA was manually extracted from 0.25 g of wet mass using a commercially available kit following manufacturer’s recommendations and eluted in 100 μL of diethylpyrocarbonate (DEPC)-treated water (Qiagen DNeasy Power Soil Kit, cat # 12888–50). Each 12 μL reaction contained 6 μL SYBR master mix (Applied Biosystems SYBR Green PCR Master Mix, cat # 4309155), 0.48 μL of a primer-water mixture (primers at final concentration of 400 nM), 4.52 μL of DEPC-treated water, and 1 μL of extracted DNA. qPCR was performed using an automated fluorometer (ABI PRISM 7900 HTA FAST, Thermo Fisher Scientific). Standard amplification conditions were used: 95°C for 3 min, 40 cycles of 95°C for 15 s, 52°C for 30 s, and 72°C for 30 s, with a melting curve at 95°C for 15 s, 52°C for 15 s, and 95°C for 15 s. Data was analyzed using Applied Biosystems SDS software, version 2.4. Fluorescent signals were collected during the annealing phase and C_q_ values extracted with a threshold of 0.2 and baseline values of 3–10 for both *ureC* and 16S assays. Amplification specificity was verified using the dissociation temperature (T_m_) of the qPCR amplicons specific to each gene. Acceptable T_m_ ranges were determined to be +/- 2% of the positive controls. For *ureC*, the acceptable T_m_ range was 80.8°C—84.1°C and for 16S it was 81.8°C—85.2°C. Samples with detectable amplification but with T_m_’s outside of the acceptable ranges were considered false positives and were deemed negative for the gene of interest. The absolute copy numbers were also normalized in terms of volatile solids (VS) mass present in the biomineral samples.

The sensitivity of ureC-F (5’-TGGGCCTTAAAATHCAYGARGAYTGGG-3’) and ureC-R (5’-SGGTGGTGGCACACCATNANCATRTC-3’) was < 4,000 copies/qPCR reaction and the efficiency was 80.6% (R^2^ = 0.9974). Poor sensitivity and low efficiency for *ureC* is expected due to the nature of SYBR degenerative primers. The 16S assay, 338F (5’- ACTCCTACGGGAGGCAGCAG-3’) and 533R (5’ -TTACCGCGGCTGCTGGCAC-3’), yielded a sensitivity of < 10 copies/qPCR reaction and efficiency of 102.2% (R^2^ = 0.9981).

### 16S rRNA Illumina high throughput sequencing

The microbial community was assessed using high throughput 16S rRNA sequencing using an Illumina MiSeq platform. First, the DNA was extracted from biomineral and liquid samples using Qiagen DNeasy PowerSoil kits following the manufacturer’s instructions. Primers 319F (TCGTCGGCAGCGTCAGATGTGTATAAGAGACAG(spacer)GTACTCCTACGGGAGGCAGCAGT) and 806R (GTCTCGTGGGCTCGGAGATGTGTATAAGAGACAG(spacer)CCGGACTACNVGGGTWTCTAAT were used to amplify the V3-V4 domain of the 16S rRNA using a twostep PCR procedure. The ureC-F and ureC-R primers used were the same as those described in the previous section.

In step one of the amplification procedure, both forward and reverse primers contained an Illumina tag sequence, a variable length spacer (no spacer, C, TC, or ATC for 319F; no spacer, G, TG, ATG for 806R) to increase diversity and improve the quality of the sequencing run, a linker sequence, and the 16S target sequence. Each 25 mL PCR reaction contained 1 Unit Kapa2G Robust Hot Start Polymerase (Kapa Biosystems), 1.5 mM MgCl_2_, 0.2 mM final concentration dNTP mix, 0.2 mM final concentration of each primer and 1 uL of DNA for each sample. PCR conditions for 16S rRNA amplification were: an initial incubation at 95°C for 3 min, followed by 25 cycles of 95°C for 45 s, 50°C for 30 s, 72°C for 30 s and a final extension of 72°C for 3 min. In step two, each sample was barcoded with a unique forward and reverse barcode combination using forward primers (AATGATACGGCGACCACCGAGATCTACACNNNNNNNNTCGTCGGCAGCGTC) with an Illumina P5 adapter sequence, a unique 8 nt barcode (N), a partial matching sequence of the forward adapter used in step one, and reverse primers (CAAGCAGAAGACGGCATACGAGATNNNNNNNNGTCTCGTGGGCTCGG) with an Illumina P7 adapter sequence, unique 8 nt barcode (N), and a partial matching sequence of the reverse adapter used in step one. The PCR reaction in step two contained 1 Unit Kapa2G Robust Hot Start Polymerase (Kapa Biosystems), 1.5 mM MgCl_2_, 0.2 mM final concentration dNTP mix, 0.2 mM final concentration of each uniquely barcoded primer and 1 uL of the product from the PCR reaction in step one diluted at a 10:1 ratio in water. PCR conditions were: an initial incubation at 95°C for 3 min, followed by 9 cycles of 95°C for 30 s, 58°C for 30 s, 72°C for 30 s and a final extension of 72°C for 3 min.

The final product was quantified on the Qubit instrument using the Qubit Broad Range DNA kit (Invitrogen) and individual amplicons were pooled in equal concentrations. The pooled library was cleaned utilizing Ampure XP beads (Beckman Coulter) then the band of interest was further subjected to isolation via gel electrophoresis on a 1.5% Blue Pippin HT gel (Sage Science). The library was quantified via qPCR followed by 300-bp paired-end sequencing using an Illumina MiSeq instrument in the Genome Center DNA Technologies Core, University of California, Davis.

### Bioinformatics and statistical analyses

All statistical work and data visualization was done using R version 4.0.2. Sequenced reads were demultiplexed and primers were trimmed using dbcAmplicons [25]. The fastq files were processed using DADA2 using the parameters described in the referenced workflow [26, 27].The protocol was modified to accommodate a new truncation length (truncLen) argument of 265 for forward and 165 for reverse reads.

For all post-sequence processing, overlapped reads were denoised, summarized to amplicon sequence variants (ASVs), and filtered for chimeric sequences using DADA2. Taxonomic assignment was done using the DADA2 implementation of the naive Bayesian classifier method using the Silva SSU database version 138 [28]. Samples with less than 10,000 reads were filtered from further analysis. Rarefaction curves were calculated for each sample using vegan and plotted using ggplot2. Various other exploratory plots involving diversity metrics were produced using phyloseq and then plotted with ggplot2 [29, 30].

Taxa diversity and evenness were assessed by calculating the Shannon index, Chao1, and observed richness using phyloseq as was taxa bar plot percentages. PERMANOVA (permutational multiple analysis of variance) was performed to compare the total sum squared dissimilarities among objects belonging to different groups. PERMANOVA testing was performed whereby demultiplexed reads were also screened for PhiX sequences, overlapped, screened for reads containing Illumina adapter sequences, filtered to exclude any reads containing uncalled bases, and filtered for a minimum overlapped length of 380 bp using HTStream [31]. PERMANOVA analyses were conducted using the function adonis2 in the R package vegan, version 2.5–6 and the function adonis.II in the R package RVAideMemoire, version 0.9–78. These analyses were based on Bray distances between log-transformed relative log expression (RLE) normalized taxon counts.

To understand the effect of geographic distances and environmental variables on the bacterial community within biomineral samples, a Mantel test was performed [32]. The environmental parameter distance matrix was generated using a Euclidean distance, the geographic coordinates matrix using the Haversine distance, and the community compositional differences using the Bray-Curtis distance. Mantel tests were performed to evaluate Spearman rank correlations between each two distance matrices or between one single factor and a matrix. Finally, non-metric dimensional scaling (NMDS) using Bray-Curtis dissimilarity was performed to visualize the community differences between samples grouped by intrasystem sampling location, seasonality, and or urinal type. Sequencing reads were deposited to NCBI’s sequencing read archives under the BioProject accession number PRJNA699694.

## Results and discussion

After the DADA2 pipeline, the microbial diversity assessment of 169 biomineral and liquid samples obtained from 11 different California state-owned rest areas resulted in a total of 3869 reference sequences aggregated as 598 OTUs. There were a total of 10,454,960 raw reads from which yielded 8,791,330 filtered reads and 8,550,615 merged reads. Of the filtered reads, 7.6% were chimeras and were removed. After DADA2 filtering, the average read lengths were 422 bp. Approximately 76.5% of the 598 OTUs were classified at the genus level, 92% at the family level, and 100% at the phylum level. However, that 23.5% remains unclassified at the genus level points to the importance of future isolation and characterization studies of microbes from urine drainage and other sanitation systems.

### The influence of sample type, urinal type, and sampling season on alpha diversity scores

**Fig 2:**
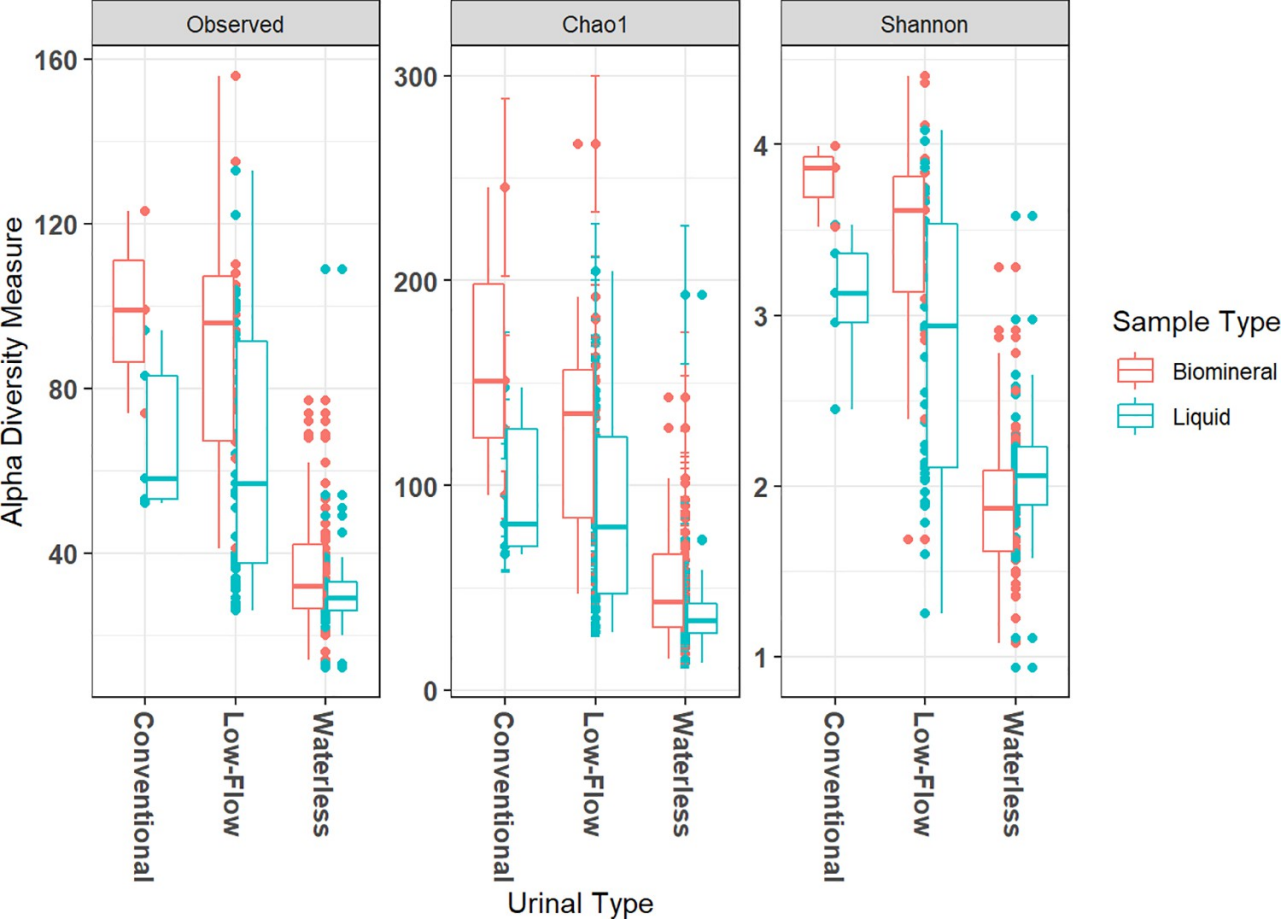
Alpha diversity metrics of all biomineral and liquid associated bacterial samples grouped by urinal type.

Alpha diversity scores within samples grouped by urinal type and by sample type (biomineral and liquid) are summarized in Fig 2. Alpha diversity describes the diversity that exists within an ecosystem and is measured by the number of species or the richness of OTUs within that ecosystem [33]. Non-parametric Kruskal-Wallis testing suggests that the alpha diversity Shannon indices for biomineral and liquid samples grouped by sample type (biomineral and liquid) do not significantly differ (*p* = 0.072). This indicates that sample type may not be significant influencer of within-sample diversity. Kruskal-Wallis testing also showed that the within-sample diversity grouped by sampling seasons differed significantly for both biomineral and liquid samples (*p* < 0.001).

While the sample size of conventional urinals is too small to draw conclusions from, we found that waterless urinals generally exhibit a lower alpha diversity score than does low-flow urinals in terms of observed richness, Chao1, and Shannon indices as shown in Fig 2. Kruskal-Wallis and pairwise Wilcoxon rank sum testing suggests that waterless urinal alpha diversity scores depart from the other two urinal types (*p* < 0.001). Our observations on lower relative Shannon indices for waterless urinals as shown in Fig 2 may be because highly ureolytic and alkaline environments characteristic of waterless urinals are more selective of alkaliphilic bacteria that can survive a high ammonia and pH environment [34–36]. This is reasonable as ammonia, which is a product of ureolysis, is known for its cytotoxic effects on bacteria at sufficiently high concentrations [35, 37]. A rapid increase in pH and ammonia may also be preventing a diverse bacterial community to develop as existing communities may not be given sufficient time to acclimate [12]. Similar diversity studies by Lahr et al. (2016) confirmed that low species richness is associated with high pH (> 9) and ammonia (> 5 g/L) characteristic of aged, hydrolyzed urine similar to the actively ureolytic environment found in urine drainage systems [34]. Others noted that ureolytic stimulation similarly decreased the community diversity in soil studies, likely due to the same pH selectivity mechanisms [12]. Previous researchers demonstrated that dilution rate is a key factor in reducing pathogenic microorganisms in urine, as high ammonia concentrations associated with waterless urinals may be correlated with greater inactivation (un-survivability) [37, 38]. The decrease in diversity demonstrated in Fig 2 may coincide with a decrease in pathogenic microorganisms found in concentrated urine.

### Waterless urinals are the least diverse of the urinal types but likely have the largest ureolytic bacterial community

**Fig 3:**
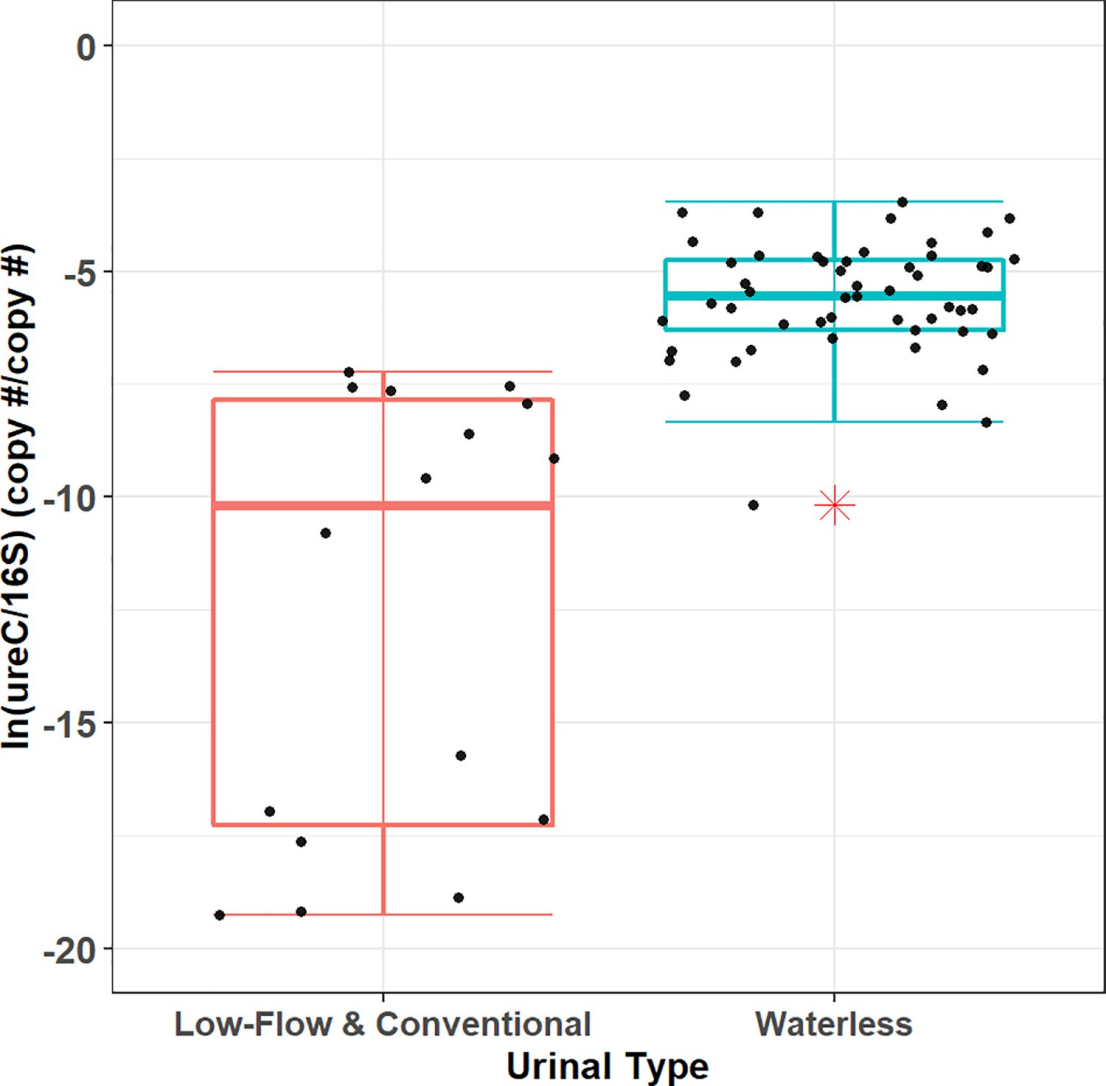
Natural logarithmic transformations of *ureC*/16S gene ratios grouped by urinal type. Low-flow and conventional urinal measurements were aggregated as one group in a comparison with waterless biomineral natural log transformed *ureC*/16S copy number ratios.

Based on the results shown in Fig 2, waterless urinal samples are the least diverse of the urinal types; however, biomineral samples obtained from waterless urinal fixtures exhibit the greatest *ureC*/16S gene copy ratios compared to those obtained from flush-type urinals. The mean *ureC* copy number per g VS is 6.22x10^7^ for waterless urinal biomineral samples and is 1.95x10^6^ for those from flush-type urinals. The mean 16S rRNA (copy number per g VS) is 9.97x10^9^ for waterless urinal biomineral samples and is 6.06x10^9^ for flush-type urinals. A t-test on the normalized, natural logarithmically transformed data shown in Fig 3 shows that the *ureC*/16S rRNA gene copy ratios grouped by urinal type differed significantly (*p* < 0.001). The large relative abundances of the *ureC* gene in waterless urinal samples suggest a possibility that there is a greater relative abundance of a potentially ureolytic community in waterless urinal samples than in low-flow samples. High concentrations of urea in concentrated urine collected from waterless urinals could lead to highly ammoniacal and alkaline conditions that select for a bacterial community exhibiting greater abundances of the *ureC* gene. The selection of the *ureC* gene due to urea supplementation has been previously reported in a cow rumen study [23]. We hypothesize that a similar phenomenon is occurring in the waterless urinals observed in this study and that there is also an environmental selection of bacteria that have the *ureC* gene. Others found that nitrogen metabolizing bacteria such as ammonia-oxidizing bacteria (AOB) increased not only in absolute numbers but also in relation to other bacterial groups investigated in urea-amended plots [39]. The relationship between ammonia concentrations in urine drainage systems and nitrogen metabolizing bacteria can be verified by future sequencing and microbial ecology studies targeting the *ureC* gene and other genes driving the microbial nitrogen cycle by building upon past studies in ureolytic environments [23, 24, 40, 41].

### Low correlations between alpha diversity and biomineral urease activity

The activity of urease was of particular interest due to its role in biomineralization. A lack of correlation between diversity scores suggests that richness and evenness alone cannot linearly predict the ammonia producing capacity associated with a given biomineral sample. Simple linear regression between Shannon indices and biomineral ureolytic activity yielded a poor goodness of fit as shown in S3 Fig from the S1 File (R^2^ = 0.014). Therefore, the community composition may not be a strong predictor of the urease activity associated with a given biomineral sample, even if there may be differences in the abundances of the potentially ureolytic community. The differences in abundances are indicated by proxy of the *ureC*/16S gene ratios for samples grouped by urinal type. Cumulatively and in direct continuity of our previous study focusing solely on the effects of environmental variables from the same sampling periods on biomineral urease activity, we conclude that the abundances of a ureolytic bacterial community and the within-sample diversity does not strongly influence biomineral activity. Our previous study suggests through a multiple regression analysis that an increase in *ureC* gene abundances does not necessarily suggest stronger urease activities [18].

In addition to quantifying gene abundances as done in this study, future studies should assess whether the transcriptional activity of *ureC* gene is influenced by urinal type and intersystem sampling locations using reverse transcriptase quantitative polymerase chain reaction (RT-qPCR) to quantify biomineral *ureC* mRNA levels. Quantifying the mRNA transcripts can provide insight into how the ureolytic community may be upregulating *ureC* transcriptional activities to produce more urease in response to differing conditions specific to the habitat. Future microbial ecology studies on urine drainpipe environments should also monitor nutrient concentrations to determine community structure relationships with mRNA transcripts.

### Microbial community composition in biomineral samples vary by urinal type

**Fig 4:**
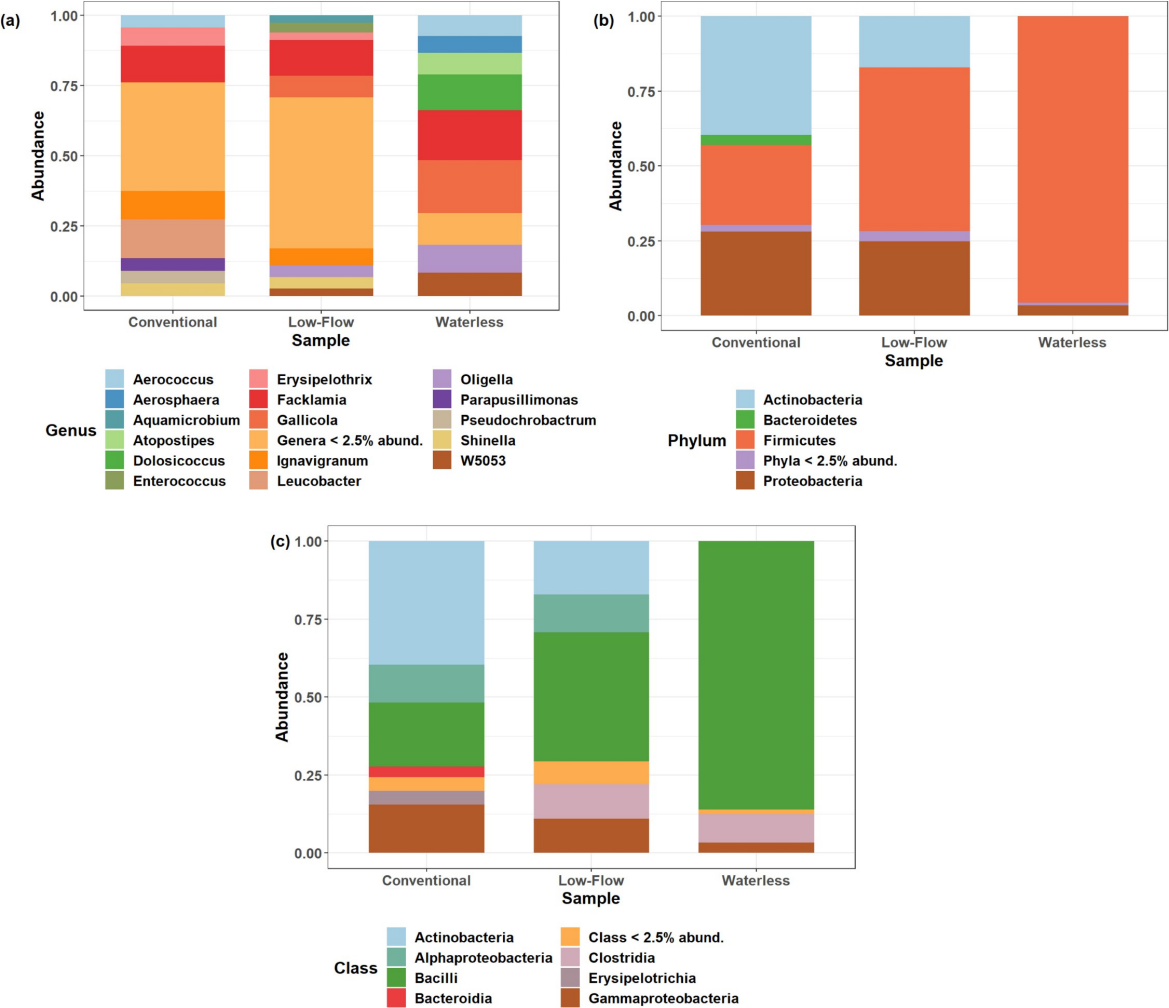
Taxonomic relative abundance bar charts grouped by urinal type. Bar charts showing relative abundances of the bacterial community grouped by urinal type are depicted at the (a) genus, (b) phylum, and (c) class level. Results were summed and averaged for all samples respective of their urinal types.

Fig 4 summarizes the abundances of taxa at the phylum, class, and genus level associated with the biomineral samples. These bar plots agree with the decrease in the alpha diversity scores based on the amount of flush water associated with each urinal type. Fig 4 suggests that the composition of conventional and low-flow biomineral samples are more similar to each other than either are to waterless biomineral samples.

At the phylum level, Firmicutes dominated the compositional structure for waterless urinals and accounted for 95.6% of all OTUs, whereas 3.4% were Proteobacteria and the remaining 1% of phyla were rare (< 2.5% relative abundance). Conversely, low-flow urinals are more diverse and were composed of 17% Actinobacteria, 54.7% Firmicutes, 24.8% Proteobacteria, and the remaining 3.5% phyla exhibited less than 2.5% relative abundance. In conventional urinals, the relative abundances of 39.6% Actinobacteria, 28.1% Proteobacteria, 26.7% Firmicutes, and 3.5% Bacteroidetes were observed. The dominance of Firmicutes in the waterless urinal biomineral samples is consistent with past metagenomic studies on saline and alkaline soils, which is sensible as the urinal environment is expected to be more saline and alkaline compared to that of conventional urinals [42]. The pluralities of Firmicutes, Actinobacteria, and Proteobacteria in samples obtained from conventional and low-flow urinals also match observations made in past restroom microbial ecology studies [43]. Firmicutes and Bacteroidetes have been reported to have a key role in organics hydrolysis and in secondary fermentation, and may thrive in environments with organic content such as the undiluted urine found in waterless urinals [44]. Though it is unclear why Firmicutes can tolerate high ammonia concentrations, that Firmicutes can may explain why they encompass the largest fraction of the community in waterless urinals, which have more concentrated urine and thus more ammonia when compared to flush-type urinals [45–47]. In biogas reactors, Firmicutes dominated the community composition in environments with total ammoniacal nitrogen concentrations (TAN) ranging from 2.4 to 4.2 g/L [47].

At the class level, waterless urinals also exhibited an increase in relative abundances of the Bacilli (86.1%) class compared to that found in low-flow (41.3%) and conventional (20.5%) biomineral samples. The presence of Bacilli has been previously observed in ureolytic microbially induced calcite precipitation soil studies [12]. Gat et al. (2016) cites Burbank et al. (2012) where it was found that 7 of 10 isolates of indigenous ureolytic soil bacteria were classified as Bacilli, which are members of the Firmicutes phyla known to be ammonia resistant as mentioned previously [48]. Gat et al. (2016) also found that a significant increase in Bacilli following ureolytic treatment coincided with an increase in culture media pH. It can then be inferred that greater urease activities observed in waterless urinal biomineral samples in our past regression study also coincide with the abundance of Bacilli bacteria [18]. Future studies can include high throughput Illumina sequencing of the *ureC* gene to determine the relative abundances of various taxa containing the gene.

Conventional urinal biomineral samples have bacterial communities distinct from both low-flow and waterless urinal samples, but the availability of ureolytic biomineralization present in the drain lines demonstrates that biomineral formation can occur in all urinal types despite differences in bacterial communities. Of the three urinal types, conventional urinals have the lowest relative abundance of Firmicutes (26.6%), but the greatest percentage of Actinobacteria (39.6%). It is possible that where Bacilli drives biomineralization in low-flow and waterless urinals, Actinobacteria could be the driver of precipitation in conventional urinals. In other ureolytic biomineralization studies on cave moonmilk formation, researchers have proposed that Actinobacteria promotes calcium carbonate precipitation by creating locally favorable conditions with the bacterial cell walls serving as the crystal nucleation sites [11, 49]. That ureolytic organisms and biomineralization found in conventional urinals persist as they do in waterless urinals suggests that increasing flush water volumes may not be enough to prevent ureolytic pipe fouling. Future studies should compare the composition of the biomineralization between each urinal type and determine the influence of the bacterial community on the mineral composition.

Cumulatively, our results confirm that restrooms host a diverse microbial community [43]. We found that the relative abundance of rare OTUs (< 2.5% relative abundance) at the genus level is 11.5% for waterless urinals compared to that observed for conventional (48.7%) and low-flow (53.9%) urinals. The abundances and richness of rare OTUs is consistent with the alpha diversity hypothesis testing results. This marked difference between the abundances of rare OTUs found in waterless and flush-type urinals is likely attributed to the differences in ammonia concentrations imparted by flush water dilution. As discussed previously, elevated ammonia concentrations and pH may lead to more a selective environment in waterless urinals where the selected alkaliphiles can tolerate elevated pH and ammonia levels. Of note, several of the genera observed can be ureolytic, but this is inconclusive unless species level identification is obtained. These genera may include *Shinella*, *Morganella*, *Tissierella*, *Thauera*, *Parapusillimonas*, *Pseudomonas*, among the more commonly observed *Oligella* [50–54]. Additionally, some genera identified in this study could be potentially pathogenic. These include genera such as *Morganella*, *Tissierella*, *Erysipelothrix*, *Atopistipes*, and *Facklamia* [51, 55–57]. Past research is consistent with our observations that potentially pathogenic genera are present in most conventional, low-flow, and waterless urinal biomineral samples [34]. Through our findings, we concur with Lahr et al. (2016), who highlighted the limitations of solely relying on enteric indicator organisms to assess bacterial risks involved in freshly (< 24 hours) collected urine from social events [34].

It was also expected that the microbial communities present in urinals are also influenced by the surrounding environment and its users. From Fig 4, the presence of *Facklamia* and *Corynebacterium*, which are typically associated with the urinary tract, is not surprising given that all sampling took place in men’s restrooms [58]. Other observed genera such as *Enterococcus* have been identified as common colonizers of human skin and gut [43]. OTUs associated with manure, compost, and soil were also identified and include *Shinella*, *Georgenia*, *Gallicola*, *Allorhizobium*, *Thiopseudomonas*, *Leucobacter*, *Tissierella*, *Atopisiptes*, *Corynebacterium*, and *Erysipelothrix* [52, 56, 59–63]. It has also been established that microbial transport can take place by air currents. Similarly, toilet and urinal flushing propels aerosols from toilet bowls into the air, and can subsequently settle on surfaces including other fixtures in the room [43]. Given the dynamic nature of busy public restrooms, it is possible that there could be cross-contamination between microbes found on the ground and those in the fixtures.

### Beta diversity analysis demonstrates the community compositional shifts influenced by intrasystem sampling location, urinal type, sample type

**Fig 5:**
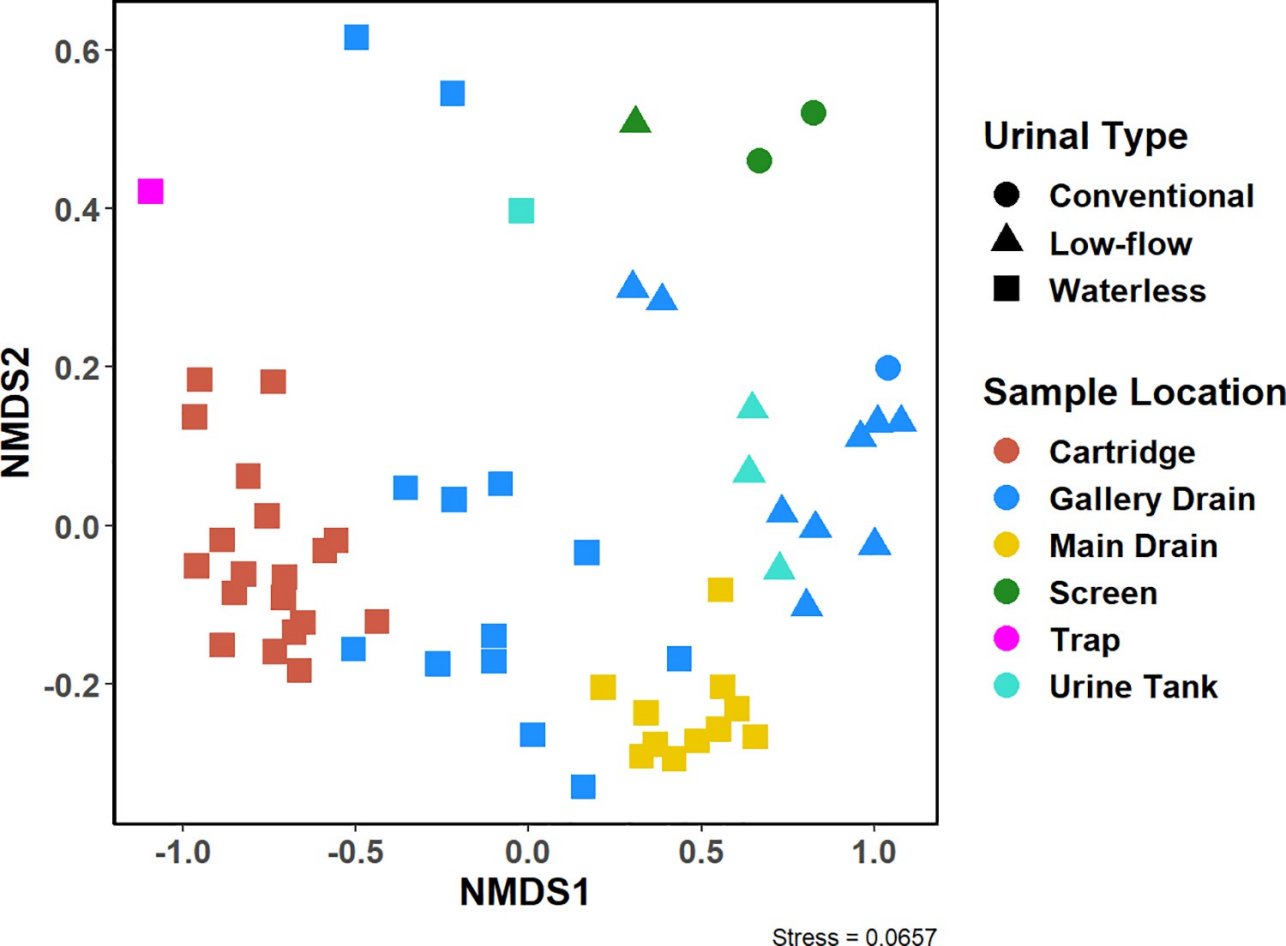
NMDS diagram of bacterial communities for all biomineral samples grouped by intrasystem sampling location.

Community (dis)similarity was estimated using the Bray-Curtis metric. From the GUSTAME multivariate statistics guide, NMDS plots can be interpreted as such [64]: objects that are ordinated closer are likely to be more similar than those further apart. The scale of the axes is arbitrary as is the ordination of the plot. Tight clusters of points that are well-separated from other clusters indicate sub-populations in the data. Fig 5 shows strong similarities between bacterial communities grouped by intrasystem sampling location indicated by strong clustering for biomineral associated communities by their respective groups. There is also suggested a goodness of fit as indicated by a low NMDS stress value of 0.06 [64]. In general, communities from gallery drain lines appear distinct from communities from waterless urinal cartridges, but some adjacent clustering is also apparent. PERMANOVA was used to test if there was a significant difference between groups of interest [64].

The community compositions significantly differ when grouped by urinal type, intrasystem sampling location, and sample type. Clustering by urinal type is apparent, whereby the bottom left side of the NMDS diagram is dominated largely by waterless samples clusters while other regions represent biomineral communities found in flush-type urinals. PERMANOVA testing for biomineral samples with at least three samples per factor also confirms that the community structure strongly differed by intrasystem sampling location (Pseudo-F = 32.56, *p* = 0.001) and urinal type (Pseudo-F = 33.03, *p* = 0.001). There also appears to be an interaction between the two main effects (Pseudo-F = 32.67, *p* = 0.001). This interaction may indicate that the observed effects on biomineral microbial communities from sampling locations depend on the urinal type. From Fig 5, a pronounced shift between communities is clear when comparing the samples taken at the user interface from the waterless urinal cartridges and the main drains, which contacts urine diluted by sink water. It is possible that the presence of water and its effects on nutrient concentrations could be influencing the bacterial community as has been observed in past soil microbial ecology studies [65]. Further PERMANOVA tests suggest that the community structure differs when grouped by biomineral and liquid sample types (Pseudo-F = 12.16, *p* = 0.001). When also controlling for urinal type in the PERMANOVA model, there were weak interactions between sample type and urinal type as variables (Pseudo-F = 2.54, *p* = 0.019).

Our statistical test suggests that the community structures differ by sample types, but this observation disagrees with Lahr et al.’s (2016) empirical observations on the similarity of liquid and struvite associated communities. Communities may differ between the liquid and biomineral samples for several reasons [66]. For one, the biofilms present in biomineralization may confer a resilience that contributes to a localized microbial community. In dental plaque studies, researchers found that the production of an adequate amount of ammonia generating capacity in ureolytic oral biofilms is essential for the stabilization of microbial communities [67]. Li et al. (2016) demonstrated that urease positive biofilms with homogeneously distributed biomineral precipitates showed greater survival when exposed to ciprofloxacin compared to a urease negative biofilm that lacked biomineral precipitates subjected to identical conditions [68]. More so, the age of the microbial communities also differs between sample types and may affect community composition. Liquid samples found in drain traps or waterless urinal cartridges are constantly flushed out as people urinate in them. Conversely, communities found in biomineral samples are more likely established and stable as they are protected by the biofilm that prevents them from being washed away.

**Fig 6:**
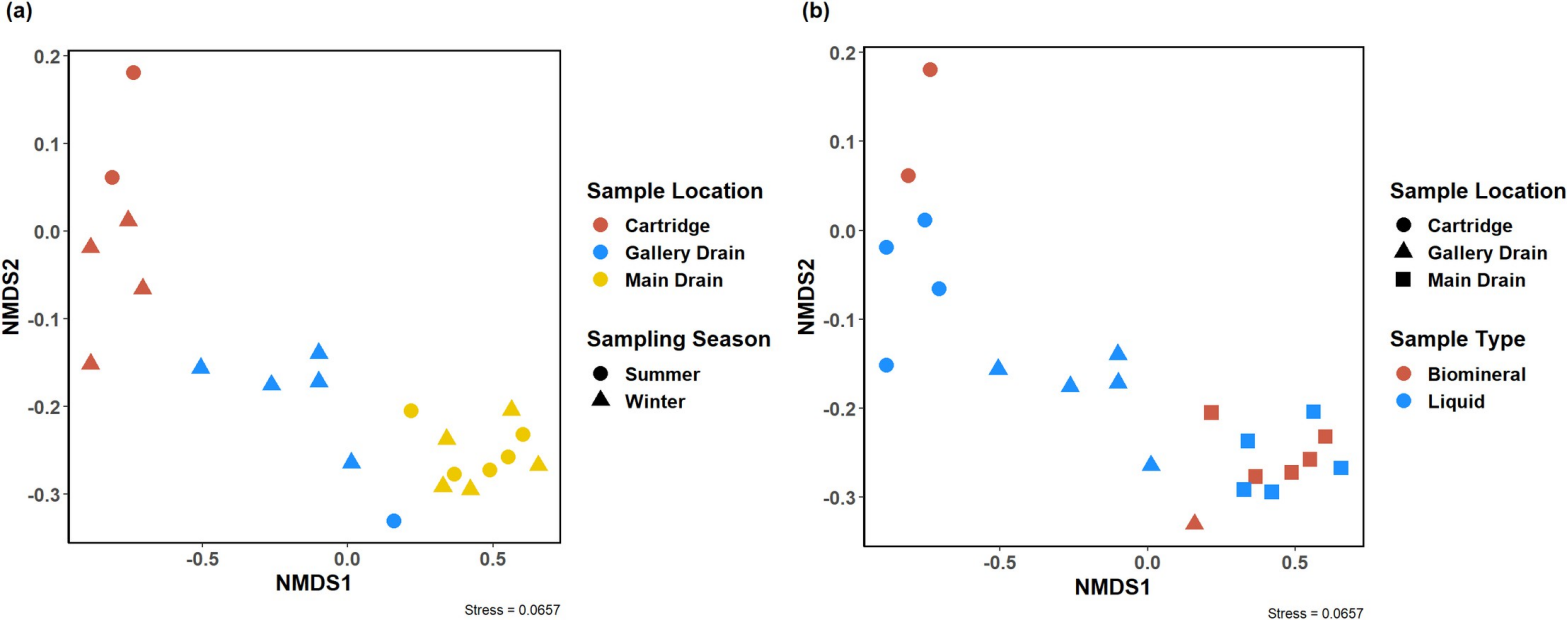
NMDS diagram of bacterial communities for biomineral samples from Westley rest area (a) grouped by sample location and sampling season, and (b) grouped by sample type and sample location.

The influence of when the samples were taken with respect to season (summer or winter) on the biomineral bacterial community structure is inconclusive—this is based on comparing PERMANOVA results using all biomineral samples and those only obtained from Westley. Based on PERMANOVA results, there were no significant large effect size differences in bacterial community composition in biomineral samples obtained from Westley between those sampled in summer and winter months (Pseudo-F = 0.973, *p* = 0.337). For Westley biomineral bacterial communities, there was also no observed interactive effects between sampling season and intrasystem sampling location (Pseudo-F = 2.06, *p* = 0.097). However, PERMANOVA testing for all sample sites including Westley suggests that the bacterial compositions can vary by sampling season (Pseudo-F = 5.26, *p* = 0.014) with interactive effects between sampling season and intrasystem sampling location (Pseudo-F = 3.59, *p* = 0.007). Community differences due to sampling location, but not sampling season are also illustrated by distinct clustering between the three sample locations in the NMDS diagram describing Westley biomineral samples (Fig 6). Notably, our previous multiple regression study suggests that sampling seasons were not a strong and significant predictor of the biomineral urease activity [18]. Because this is the first report on ureolytic biomineral communities from urine drainage systems, future microbial ecology studies on urine drainage systems should continue to describe the effects of seasonality for comparison. Inconclusive observations on the (dis)similarity between biomineral bacterial communities grouped by seasonality was unexpected, as temperature and seasonality is a common distinguishing factor between communities [69, 70].

Conversely, the biomineral bacterial communities associated with Westley (Fig 6) followed similar trends observed for all sample sites (Fig 5). Biomineral communities from Westley significantly differed based on intrasystem sampling locations within the urine drainage system (Pseudo-F = 31.56, *p* = 0.001).

### Limitations of the study

Due to cost and scheduling constraints, one limitation of this microbial ecology study is that some locations were more well-sampled than others. For example, only two conventional urinal sites were studied, but this leaves many opportunities for future microbial ecologists to explore. Having more evenness in the sampling distribution would have conferred greater statistical power when drawing conclusions about conventional urinal biomineral samples.

Moreover, certain rest areas were more well sampled than others due to the availability of biomineral samples in the pipes and urinal cartridges. Cleaning, at times, affected the availability of biomineralization in the drainpipes, and so sufficient amounts may not have been available during certain sampling days. Consider that some rest areas may have been recently cleaned by maintenance staff prior to sampling while other sites may have established biomineral microbial communities in the drain lines. Some rest areas were also designed to be more accessible than others, as clean-out ports were not installed in every plumbing gallery. This meant that obtaining biomineral samples from certain locations at each rest area may not have been feasible. In addition to making it difficult to study some of the facilities, the lack of clean-out ports is a design flaw, as it would make removing clogs difficult and costly should they occur. In the future, engineers should incorporate clean-out ports in waterless urinal plumbing fixtures where possible.

It is possible that the environmental metadata used in the NMDS was incomplete as suggested by Mantel testing. A Mantel test was used to estimate the degree of geographic distance influencing the compositional differences between various rest sites. It was found that the microbial community is more strongly correlated with geographic distance than with the environmental factors (Mantel statistic r: 0.685, *p* < 0.001). The correlations in the Haversine distance matrix suggest that geographically closer samples are compositionally more similar to each other than those further apart. This is expected as environmental conditions found in regions grouped closer together are also expected to be more similar. Mantel testing also demonstrated that the measured environmental factors are moderately correlated with biomineral microbial community (Mantel statistic r: 0.351, *p* < 0.001). Samples which are similar in terms of moisture content, biomineral ureolytic activity, *ureC* gene copies/VS (g), and VS per total solids (TS) ratios tend to be more similar in terms of their microbial community composition. It is possible that the environmental factors are less correlated to the community composition than geographic distance because the environmental dataset is incomplete. Had we included pH, nutrient, and ion concentrations in our study, then there could have appeared stronger correlations between environmental variables and the community composition indicated by the r value in the Mantel test.

Finally, the greater influence by sampling sites on community composition than the environmental variables measured in this study indicates another possible limitation of the study. Because rest areas are highly dynamic environments, there are uncontrolled factors such as differences in cleaning products as well as the frequency and intensity of cleaning performed at each rest area. Differences in cleaning procedures and frequency can also affect the age of the biomineral samples within the pipes and cartridges. Subsequently, the age of the samples was uncontrolled in the study, which could affect the physical and community composition of the samples. Though controlling for sample sage would be ideal, we note that it would have been impractical to impose a strict cleaning schedule on rest area laborers statewide. Future studies on the effects of biomineralization age on the microbial community structure and on urease activity should be conducted.

## Supporting information

S1 File Supporting information. (PDF)
