# Supplementary material for: A biogeographic 16S rRNA survey of bacterial communities of ureolytic biomineralization from California public restrooms
Source: PLoS One. 2022 Jan 14;17(1):e0262425. doi: 10.1371/journal.pone.0262425 (PMC8759634; doi:10.1371/journal.pone.0262425)
Supplement: S1 File — (PDF) [file pone.0262425.s001.pdf]

SUPPORTING INFORMATION

S1 File

A biogeographic 16S rRNA survey of bacterial communities of ureolytic biomineralization from California Public Restrooms

Kahui Lim [†], Matt Rolston[#], Samantha Barnum [§], Cara Wademan [§], Harold Leverenz [†]

[†] Department of Civil and Environmental Engineering, University of California at Davis

[#] Host Microbe Systems Biology Core Facility, Dept. of Medical Microbiology & Immunology

[§] Real-time PCR Research & Diagnostics Core Facility, Dept. of Medicine & Epidemiology, University of California at Davis

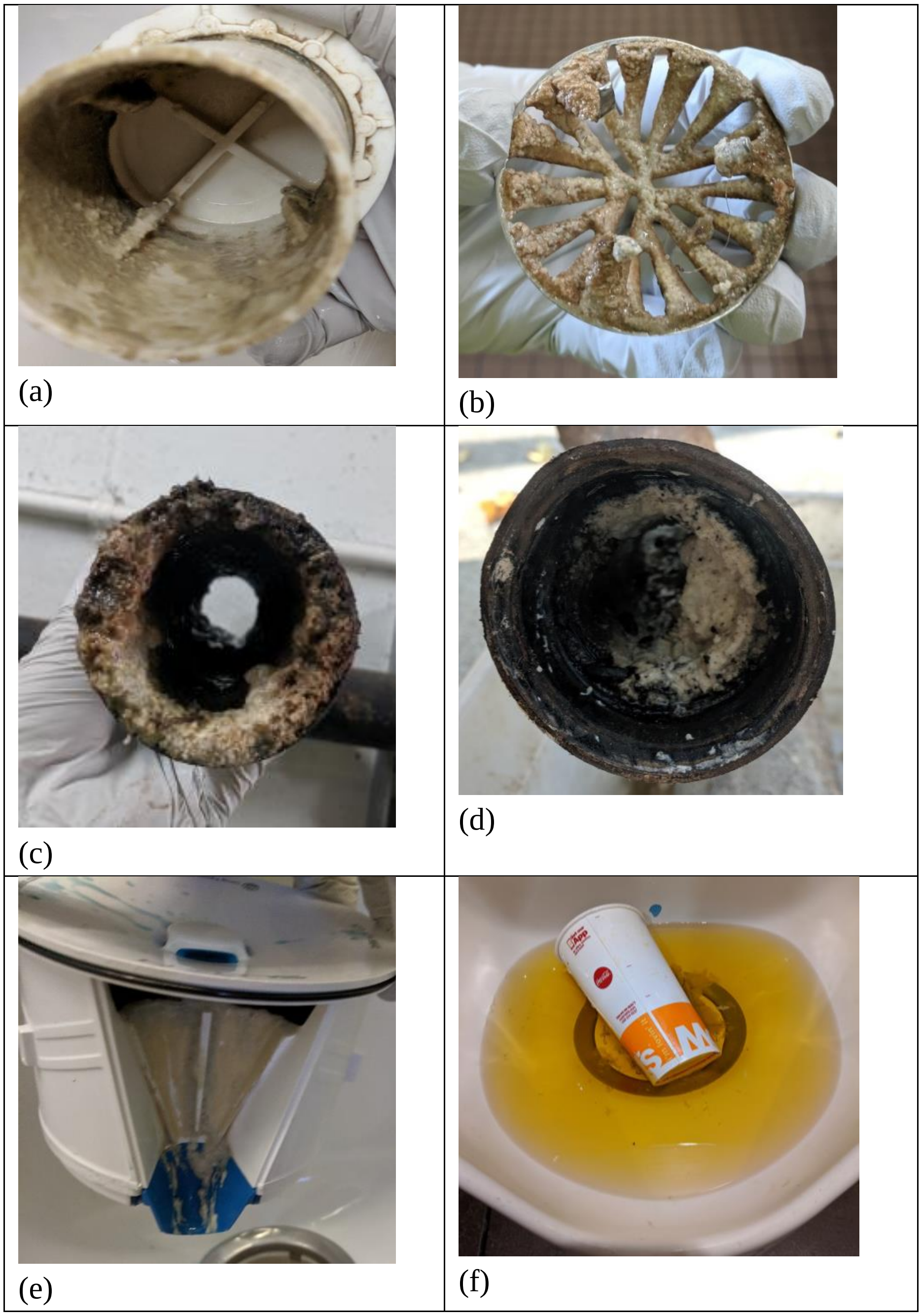

S1 Fig. Panels a-e demonstrate the extent of biomineral formation in conventional, low-flow, and waterless urinals. Any surface that urine touches is susceptible to biomineralization, including cartridges, screens, drain traps, and drainpipes. The pictures are a) biomineral formation on a cartridgeless trap design at Grass Lake on 27 Aug 2019, b) Biomineralization on a metal screen inside a urinal at RE Collier south men’s restroom on 28 Aug 2019, c) view of typical combined biomineral formation and corrosion of 2” iron pipe, d) Another view of reduced internal pipe diameter by biomineralization in urine drainage pipe at Dunnigan northbound SRRA on 12 Dec 2019, e) biomineralization formation on waterless urinal

cartridge at Erreca on 16 Sep 2019, f) Example of vandalism that leads to clogging observed at a waterless urinal in a gas station along I-5 on 12 Mar 2020.

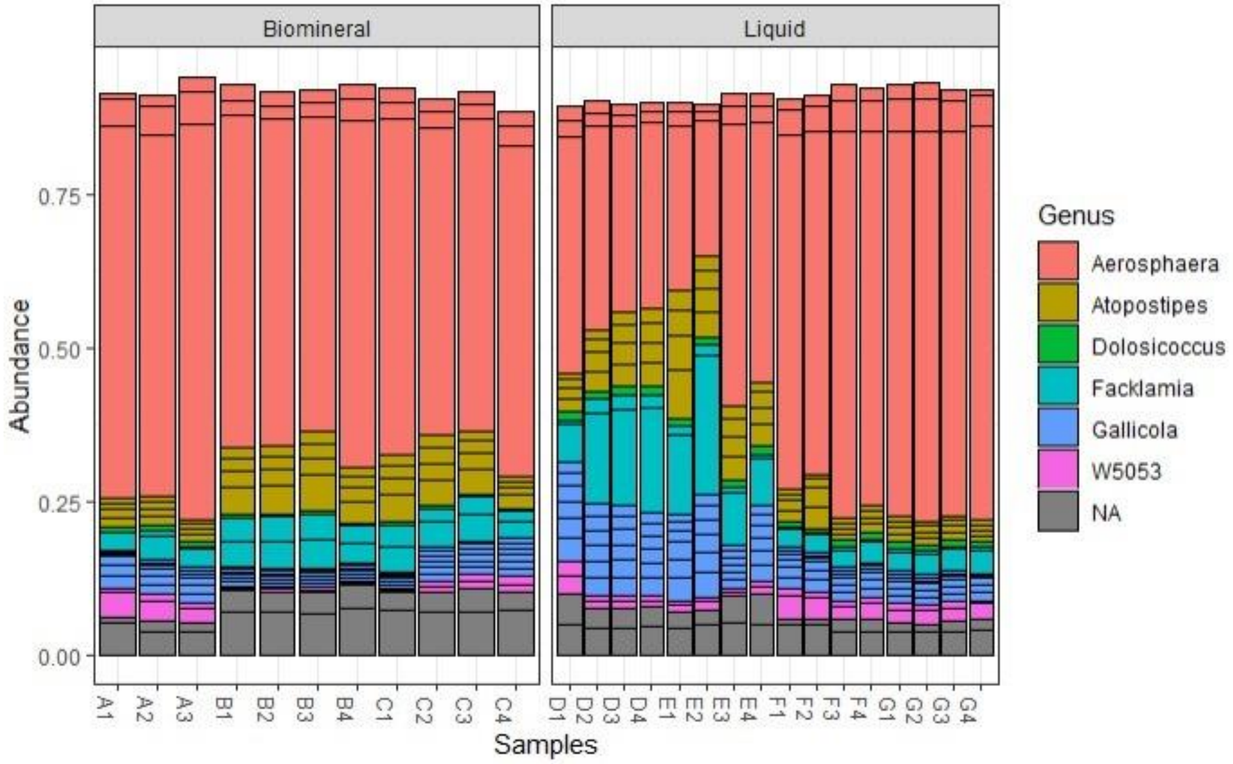

S2 Fig. An exploratory bar plot for comparing the effects of sample storage time and temperature on the outcome of high throughput sequencing is shown. Biomineral samples from Dunnigan northbound SRRA sampled from a single day were subject to various storage conditions to provide evidence that samples taken during the study were adequately stored during transportation.

Sample Group A1-3: 4ºC storage at 0, 2, 5 days prior to freezing
Sample Group B1-4: 4ºC storage at 0, 2, 5, 35 days prior to freezing
Sample Group C1-4: 21ºC storage at 0, 2, 5, 35 days prior to freezing
Sample Group D1-4: 4ºC storage at 0, 2, 5, 35 days prior to freezing
Sample Group E1-4: 21ºC storage at 0, 2, 5, 35 days prior to freezing
Sample Group F1-4: 4ºC storage at 0, 2, 5, 35 days prior to freezing
Sample Group G1-4: 21ºC storage at 0, 2, 5, 35 days prior to freezing

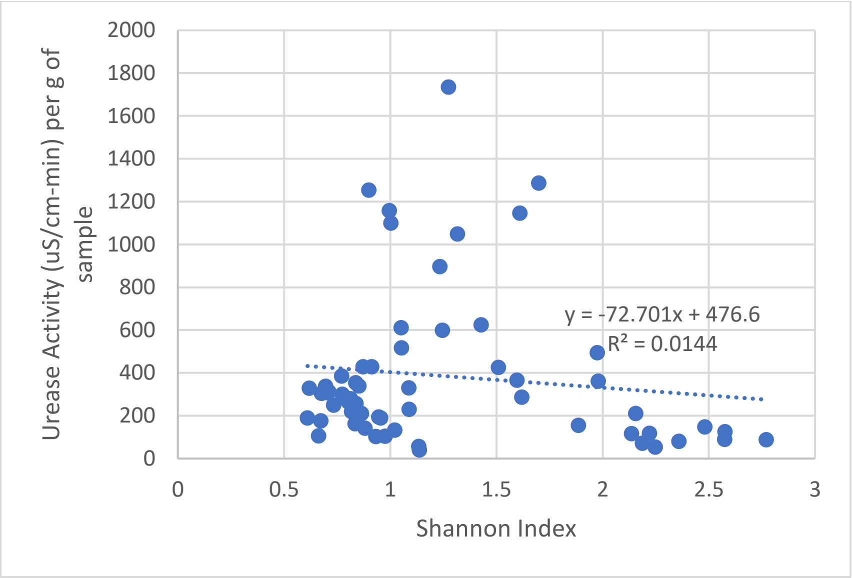

S3 Fig. Simple linear regression relating ureolytic activity with Shannon indices of biomineral samples. No correlation is observed between within-sample diversity scores and ureolytic activity indicated by the negligible coefficient of determination.
